# Periconceptional nutrition with spineless cactus (*Opuntia ficus-indica*) improves metabolomic profiles and pregnancy outcomes in sheep

**DOI:** 10.1038/s41598-021-86653-w

**Published:** 2021-03-30

**Authors:** César A. Rosales-Nieto, Maribel Rodríguez-Aguilar, Francisco Santiago-Hernandez, Venancio Cuevas-Reyes, Manuel J. Flores-Najera, Juan M. Vázquez-García, Jorge Urrutia-Morales, Morteza Hosseini Ghaffari, César A. Meza-Herrera, Antonio González-Bulnes, Graeme B. Martin

**Affiliations:** 1 Facultad de Agronomía y Veterinaria, Universidad Autónoma de San Luis Potosí, 78321 San Luis Potosí, Mexico; 2 Coordinación para la Innovación y Aplicación de la Ciencia y la Tecnología (CIACYT), Universidad Autónoma de San Luis Potosí, 78210 San Luis Potosí, Mexico; 3 Present Address: Departamento de Farmacología, División de Ciencias de la Salud, Universidad de Quintana Roo, 77039 Chetumal, Quintana Roo México; 4 Instituto Nacional de Investigaciones Forestales, Agrícolas y Pecuarias, Campo Experimental San Luis, 78431 San Luis Potosí, Mexico; 5 Instituto Nacional de Investigaciones Forestales, Agrícolas y Pecuarias, Campo Experimental Valle de México, 56250 Texcoco, Ciudad de México Mexico; 6 Instituto Nacional de Investigaciones Forestales, Agrícolas y Pecuarias, Campo Experimental la Laguna, 27440 Matamoros, Coahuila Mexico; 7 Institute of Animal Science, Physiology and Hygiene Unit, University of Bonn, 53115 Bonn, Germany; 8 Unidad Regional Universitaria de Zonas Áridas, Universidad Autónoma Chapingo, 35230 Bermejillo, Mexico; 9 Departamento de Producción y Sanidad Animal, Facultad de Veterinaria, Universidad Cardenal Herrera-CEU, CEU Universities, 46115-Alfara del Patriarca, Valencia, Spain; 10 UWA Institute of Agriculture, University of Western Australia, Crawley, WA 6009 Australia

**Keywords:** Developmental biology, Physiology, Zoology

## Abstract

We tested whether periconceptional nutrition with cladodes from the cactus, *Opuntia ficus-indica*, with or without protein-enrichment, improved the metabolomic profile and reproductive outcomes of adult female sheep. Sixty Rambouillet ewes of similar body weight were randomly allocated among three nutritional treatments that were fed during the breeding period (34 days): Control (Control; n = 20), *Opuntia* (Opuntia; n = 20) and protein-enriched *Opuntia* (E-Opuntia; n = 20). There were no effects of treatment on body weight but assessment of urine samples indicated that, for 76 metabolites, the Control and Opuntia groups differed completely (P < 0.05), whereas there was overlap between the Control and E-Opuntia groups. It appears that, in Opuntia-fed and Control-fed sheep, different functional groups are activated leading to changes in the metabolism of glucose, tyrosine, methane, and glycerolipids. Fertility and reproductive rate tended to be higher in the Opuntia (70% and 95%) and E-Opuntia (90% and 110%) groups than in the Control (55% and 65%), and an orthogonal contrast revealed the difference between Control and Opuntia to be significant for both reproductive variables (P < 0.05). We conclude that nutritional supplementation with *Opuntia* cladodes, with or without protein enrichment, increased fertility rate and reproductive rate of female sheep, without any accompanying increases in body weight. Our observations suggest that the reproductive responses to Opuntia do not simply reflect a response to good nutrition, but might be caused by specific metabolites/metabolomic pathways, perhaps by an activation of the metabolism of glucose, methane, tyrosine and glycerolipids. There are few reports relating these metabolomic compounds with the metabolism of the sheep, let alone with reproductive efficiency. The novelty of these discoveries suggests that we need further research into the mechanisms through which nutrition affects the reproductive system.

## Introduction

Small ruminants represent the principal economic output and thus contribute a large share to the income of farmers in arid and semiarid regions. These regions are often characterized by overgrazing, poor-quality soils and low annual rainfall that is also highly variable from year to year. Accordingly, the pattern of forage supply varies from year to year and crop production is unreliable^1^, and this scenario is becoming more difficult with climate change. Animals raised under these conditions generally do not receive nutritional supplements because high-quality pasture and concentrate feeds are too expensive^2^. The productivity of the system therefore depends directly on the forage provided by degraded rangelands^3^. Nevertheless, production from ruminants in these dryland systems is expected to play an increasingly important role in feeding the world, so innovative and versatile options for livestock production are needed to maximize productivity and improve ecosystem health^4^. There is a clear need for abundant, low-cost alternative feed supplements that can improve the production efficiency of animals reared under these difficult conditions.

A variety of alternative supplements have been proposed, such as betacarotene and ball moss (*Tillandsia recurvata*)^5,6^. For arid and semi-arid areas, there is considerable interest in the *Opuntia* genus, an abundant plant that can be used as a feed supplement and also seems to confer benefits for reproduction in small ruminants^7–9^. As a succulent, *Opuntia *spp. can withstand water shortage and high temperatures, and they adapt readily to poor soils, so they can be cultivated at low cost^10,11^. Importantly, the distribution of *Opuntia ficus-indica* encompasses Latin America, South Africa and the Mediterranean area^12^.

The cladodes of *Opuntia ficus-indica* are highly digestible and can provide water and energy^13,14^, although the protein content is low and varies with plant maturity, ranging from 2.8 to 4.1%^13,14^. The protein content can be improved by fermentation with additives or by adding urea^15–18^. Supplementation with *Opuntia* cladodes during specific periods of the production cycle therefore seems to be a real option for arid and semiarid conditions.

Feeding *Opuntia* cladodes can improve ovulation rate, the response to the male effect, and postpartum ovarian activity^17,19^. The consensus for the effects of improved nutrition on ovarian activity is that circulating glucose and related homeostatic hormones act directly on follicle development^20^. However, most of the studies leading to this perspective were based on experiments using supplementation with lupin grain. There is some information on the metabolic pathways activated by feeding with *Opuntia* cladodes^21,22^, but responses to protein-enriched cladodes have not been studied, for either metabolic profiles or reproductive outcomes. It is feasible that increasing the protein content of *Opuntia* cladodes will increase protein synthesis in the ovary and therefore influence follicular development and enhance fertility^20^, especially when the nutritive quality of other feed is low.

For evaluating phytochemical bioactive compounds and determining the quality of diets, metabolomic techniques have emerged as an important tool^23,24^. These techniques are now considered valuable for addressing future needs in agriculture and animal production because they can be used to measure and detect molecules in biofluids and therefore unravel the pathways leading to production of specific biomarkers. Metabolomics might therefore provide further insights into the mechanisms responsible for improvements in production and reproduction after the feeding of supplements such as *Opuntia* cladodes^25^. Using a suite of metabolomic measurements, we therefore tested the hypothesis that supplementing the diet of adult female sheep with *Opuntia* cladodes would improve the metabolic profile, increase body weight gain, and enhance reproductive performance. We also tested whether these responses would be improved if the cladodes were protein-enriched.

## Results

Experimental measurements were made Day − 14 (the first day of the 2-week adaptation period), Day 0 (the first day of dietary treatment), Day 30 of treatment, and Day 45 (10 days after the end of treatment).

### Body live weight and body live weight change

There were no significant differences in ewe body weight among treatments, in absolute values or in changes over time throughout the experiment (P > 0.05; Table 1).

**Table 1:** Ewe body weight at the start of the experiment, at the start of breeding, and at the end of breeding period, and bodyweight change during the breeding period in mature Rambouillet ewes. The animals were offered only alfalfa (Control), protein-enriched opuntia (E-Opuntia) or Opuntia during the breeding period.

Item	E-Opuntia	Opuntia	Control	SEM^a^	P-value
Starting body weight (kg)	39.5	41.0	39.6	1.96	0.48
Breeding body weight (kg)	42.2	44.5	43.1	2.15	0.29
End body weight (kg)	43.3	45.6	43.8	2.12	0.26
Bodyweight change (g/day)	34	34	23	18.9	0.65

^a^Standard error of the mean from the mixed model output.

### Fertility and reproductive rate

Fertility rate was 55% (11/20 ewes) in the Control group, 70% (14/20) in the Opuntia group, and 90% (18/20) in the E-Opuntia group. The initial statistical analysis only revealed a tendency for differences among treatments (P = 0.07), but an orthogonal contrast showed that the Control and E-Opuntia treatments differed significantly (P < 0.05). In the ewes that conceived, reproductive rate was 65% (2 pregnant ewes carrying twins out of 11 pregnant ewes) in the Control, 95% (5 pregnant ewes carrying twins out of 14 pregnant ewes) in the Opuntia group, and 110% (4 pregnant ewes carrying twins out of 18 pregnant ewes) in the E-Opuntia group. Again, none of the differences were significant (P > 0.05) except for the orthogonal contrast that revealed a significant difference between Control and E-Opuntia (P = 0.03). Body weight at the start of mating or body weight change during the mating period was not correlated with fertility or reproductive rates (P > 0.05).

### Urine metabolomic profile

**Figure 1:**
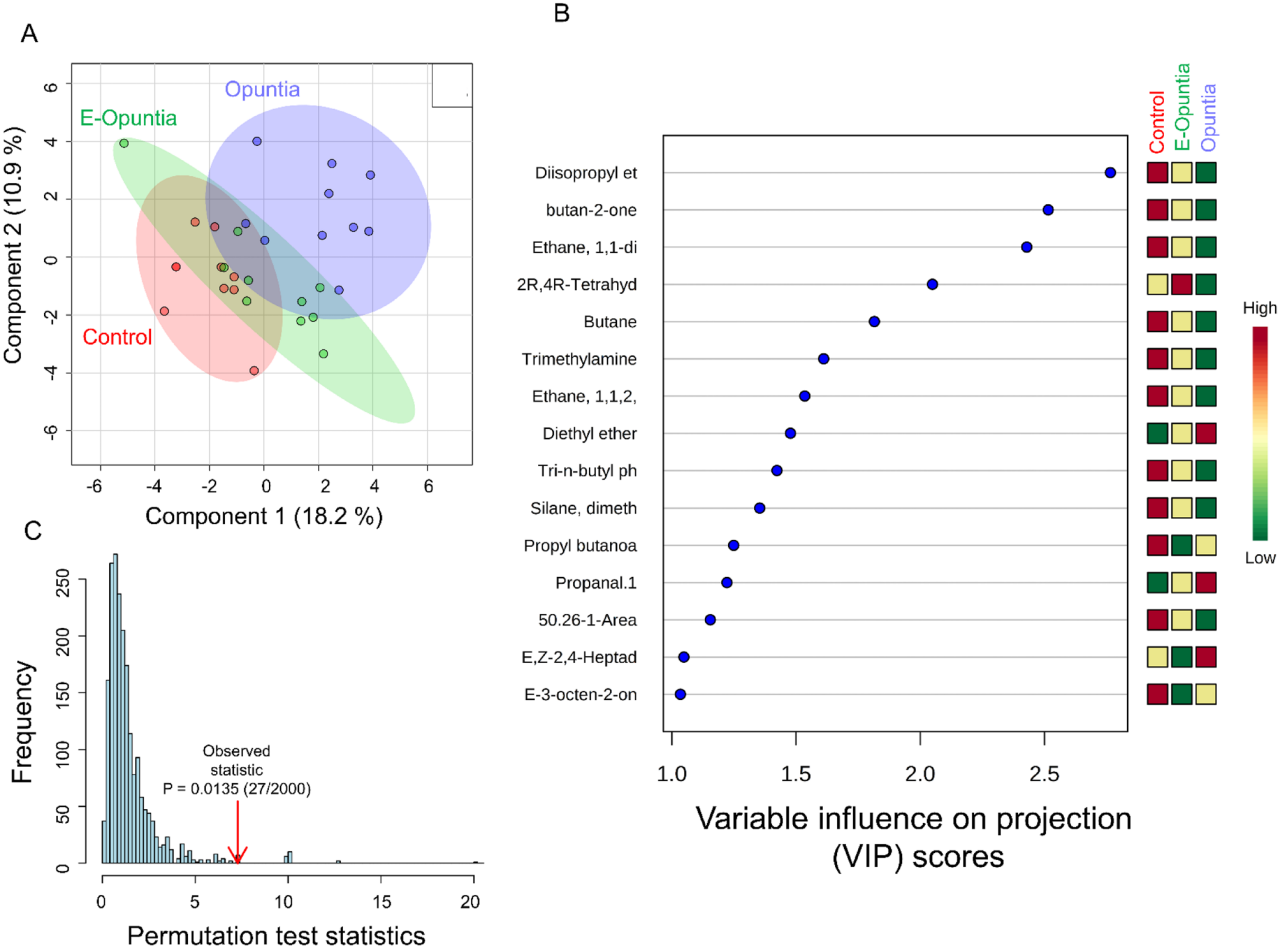
(**A**) Partial least squares-discriminant analysis (PLS-DA) showing 2 clusters for 3 treatments (Control, E-Opuntia, Opuntia); (**B**) the metabolites ranked by variable importance in projection (VIP) before the start of dietary treatments (Day 0). (**C**) PLS-DA model validation by permutation tests based on separation distance of treatments. The p-value based on permutation was P = 0.014 (27/2000). Metabolomics data analysis was performed using the free web-based metabolomics tool (MetaboAnalyst 4.0; https://www.metaboanalyst.ca/home.xhtml)^54^.

**Figure 2:**
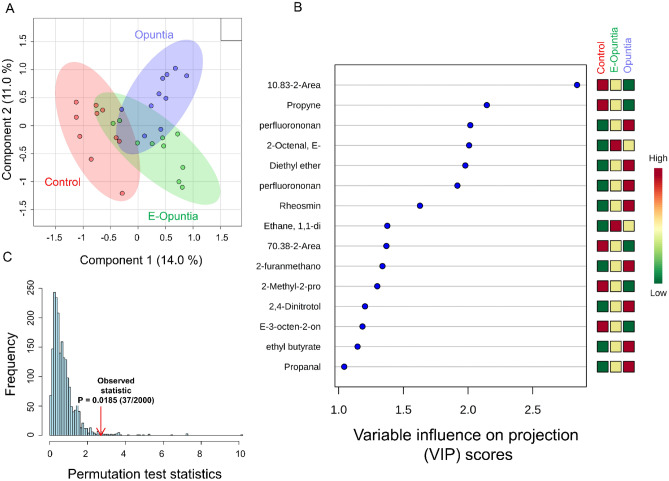
(**A**) Partial least squares-discriminant analysis (PLS-DA) showing 2 clusters for 3 treatments (Control, E-Opuntia, Opuntia); (**B**) the metabolites ranked by variable importance in projection (VIP) during dietary treatment (Day 30). (**C**) PLS-DA model validation by permutation tests based on separation distance of treatments. The p-value based on permutation was P = 0.0185 (37/2000). Metabolomics data analysis was performed using the free web-based metabolomics tool (MetaboAnalyst 4.0; https://www.metaboanalyst.ca/home.xhtml)^54^.

**Figure 3:**
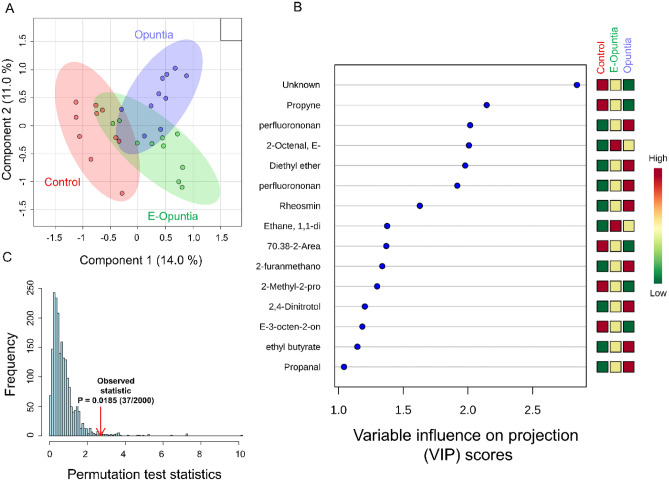
(**A**) Partial least squares-discriminant analysis (PLS-DA) showing 2 clusters for 3 treatments (Control, E-Opuntia, Opuntia); (**B**) the metabolites ranked by variable importance in projection (VIP) after the end of dietary treatment (Day 45). (**C**) PLS-DA model validation by permutation tests based on separation distance of treatments. The p-value based on permutation was P = 0.059 (119/2000). Metabolomics data analysis was performed using the free web-based metabolomics tool (MetaboAnalyst 4.0; https://www.metaboanalyst.ca/home.xhtml)^54^.

**Figure 4:**
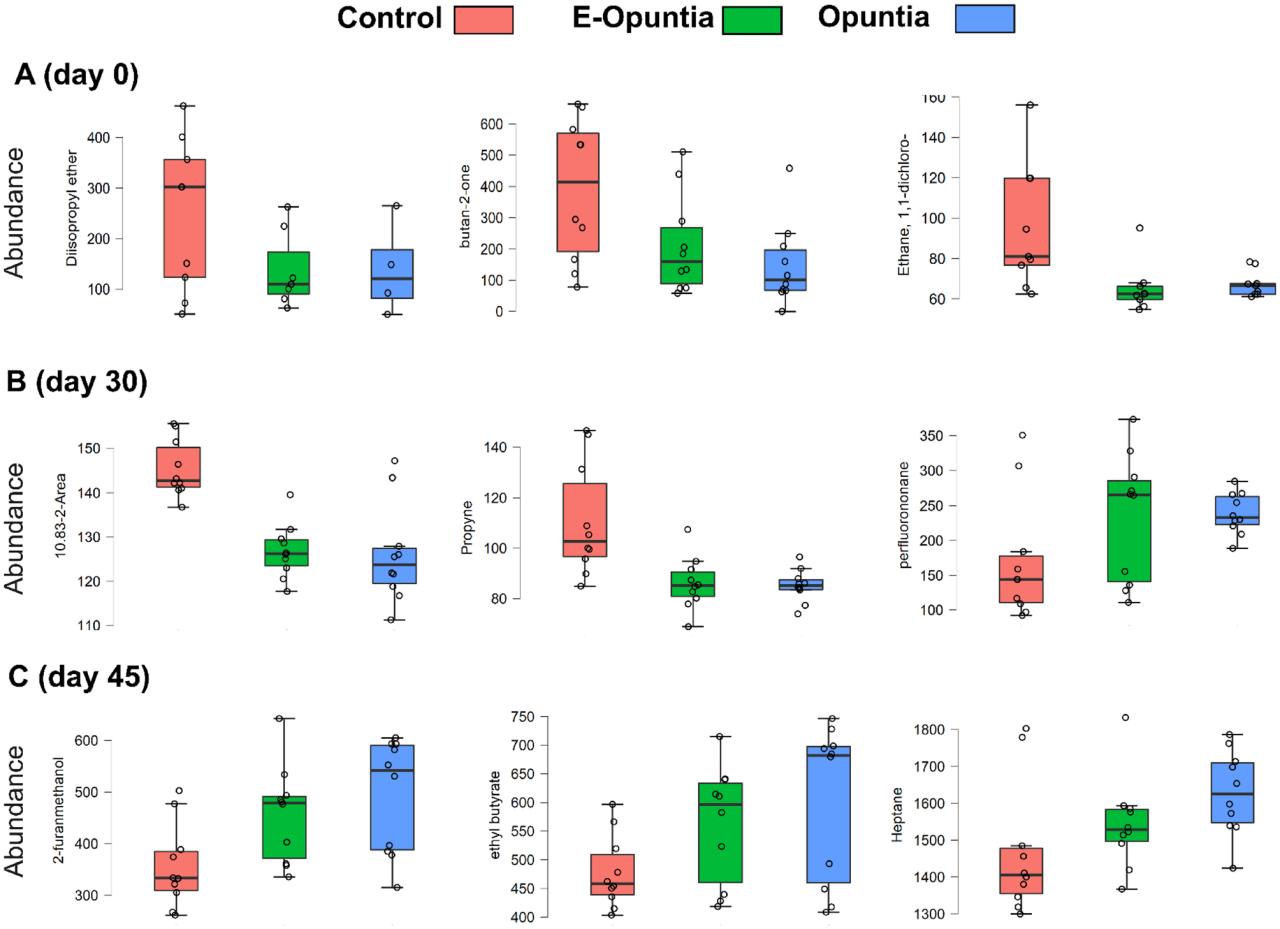
The important metabolites that contributed most significantly to the separation of treatments, according to the VIP scores using the above PLS-DA model before the start of dietary treatments. (**A**) Figure represents Day 0, (**B**) figure represents Day 30, (**C**) figure represents Day 45. Metabolomics data analysis was performed using the free web-based metabolomics tool (MetaboAnalyst 4.0; https://www.metaboanalyst.ca/home.xhtml)^54^.

On Day − 14 no difference on metabolomic profile among nutritional treatments was observed (Supplemental Figure 1); afterward, across all urine samples from all treatments, a total of 76 metabolites were consistently identified, of which 13 had indiscrimination index of 0.99 (Table 2). The PLS-DA score plots based on the urine metabolome database on Day 0 (Fig. 1A), Day 30 (Fig. 2A) and Day 45 (Fig. 3A) revealed a complete and significant separation of the Control and Opuntia treatments, but an overlap between Control and E-Opuntia (Figs. 1C, 2C, 3C). The metabolites that contributed most significantly to the separation of treatments, according to the VIP scores using the above PLS-DA model, were: three metabolites on Day 0 (diisopropyl ether, butane-2-one, and ethane; Figs. 1B and 4A), five metabolites on Day 30 (unknown metabolite, propyne, perflurononan, 2-octenal, and diethl ether; Figs. 2B and 4B) and three metabolites after the end of dietary treatment on Day 45 (2-furanmethanol, ethyl butyrate, and heptane; Figs. 3B and 4C).

**Table 2:** Urine metabolomic compounds with an indiscrimination index of 0.99.

Compound	Functional group
2-Methyl-2-propanol	Alcohol
2-Methylbutane	Ester
butane	Hydrocarbon
2-Propionylpyrrole	Ether
Heptane	Ester
Propyne	Aldehydo
Methane, bromodichloro-	Trihalomethane
Ethyl butyrate	Ester
Diethyl ether	Ether
β-ionone	Hydrocarbon
2-furanmethanol	Ether
Bis(2-methyl-3-furanyl)disulfide	Amina
2-Octenal, (E)-	Amina

### Time-resolved analysis of metabolomics data

**Figure 5:**
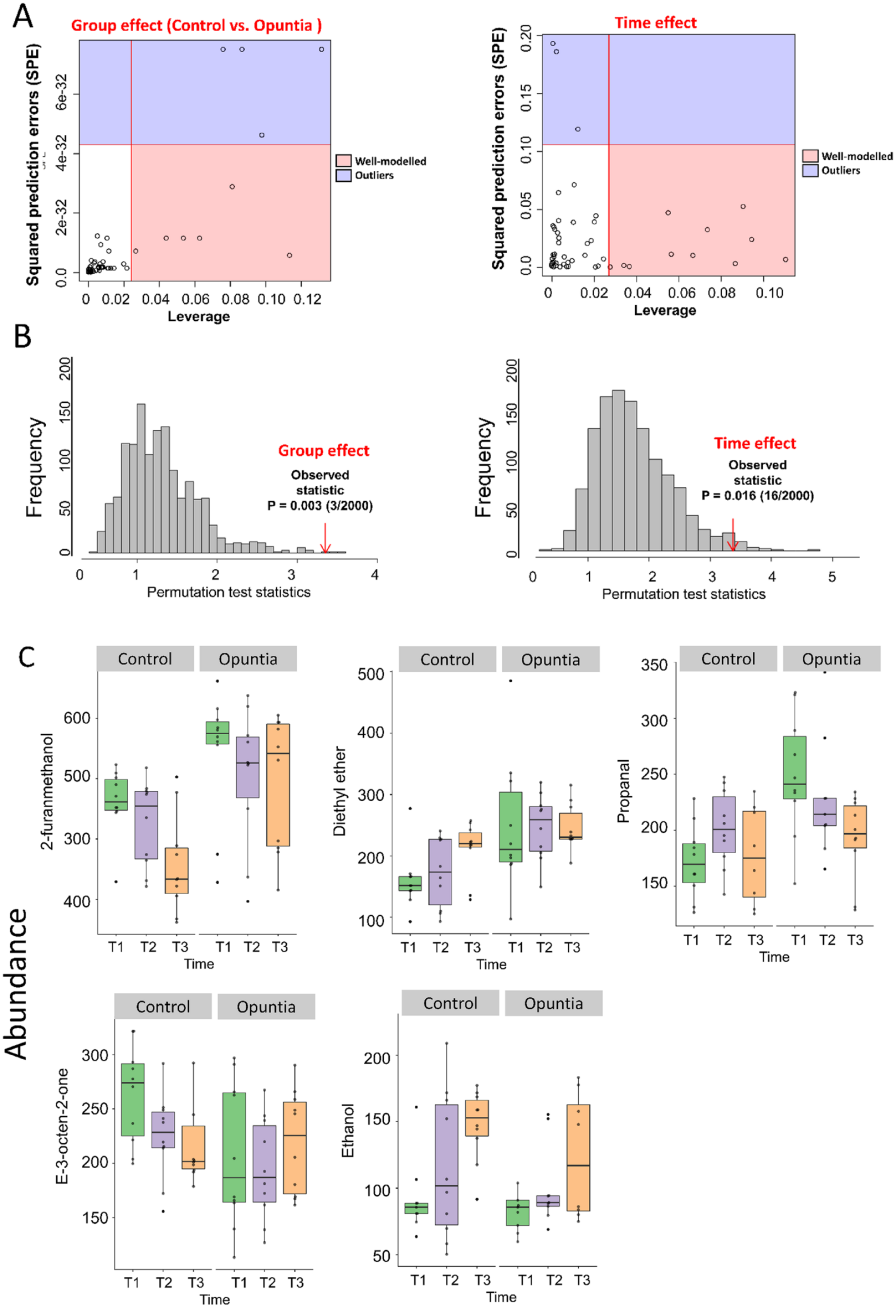

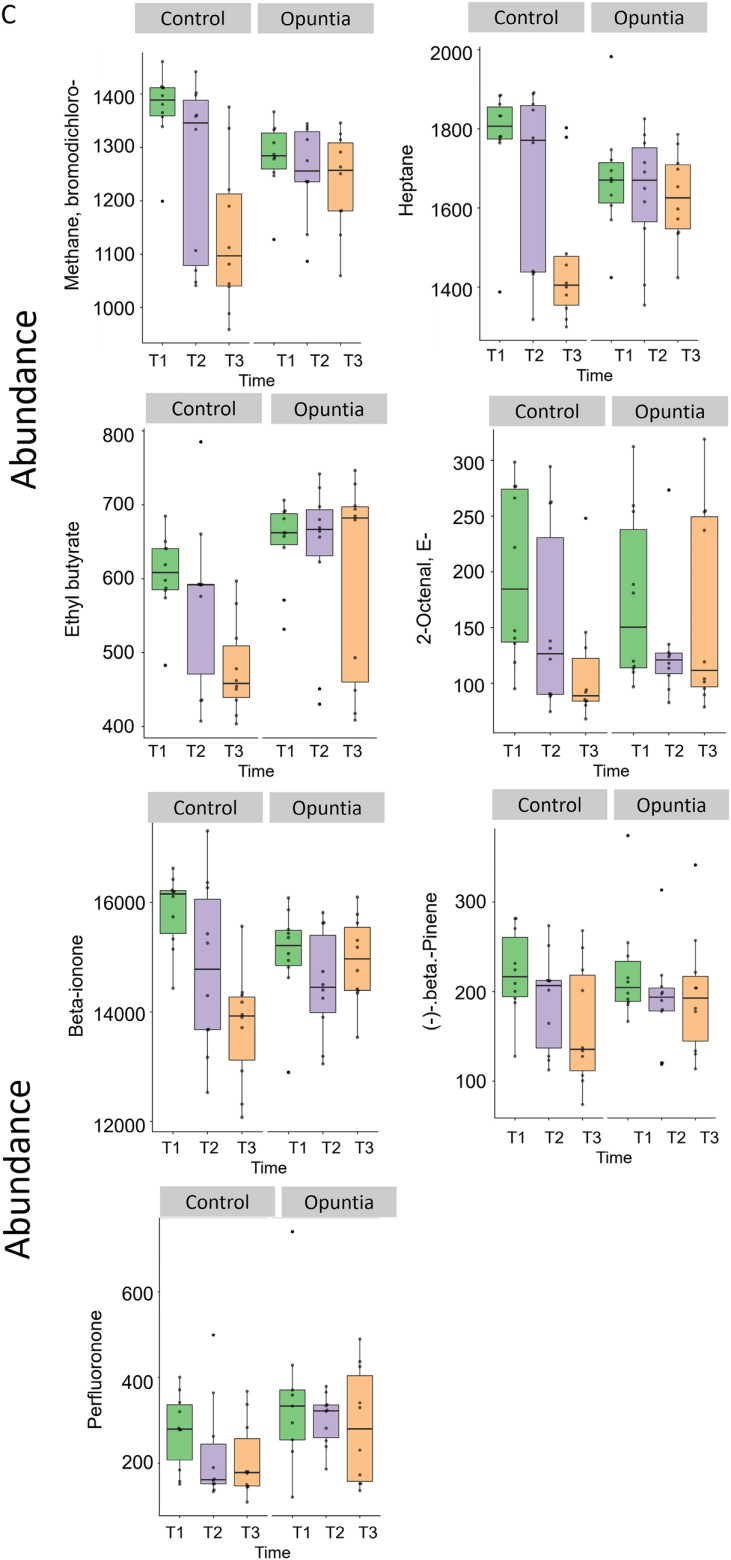
(**A**) Leverage and squared prediction error (SPE) scatter plots of the ANOVA-simultaneous component analysis (ASCA) variables sub-models for Control and Opuntia sheep, and time. Vertical and horizontal lines indicate cut-off leverage and SPE values, respectively. The leverage evaluates the importance of the metabolite to the model, and SPE tests the fitness of the model for particular metabolites. Metabolites with a high leverage value and a low SPE value were considered to be differential metabolites (the well-modelled group). Metabolites in blue have patterns that are different from the major patterns. Metabolites in the white area have patterns that are similar to the major patterns. (**B**) The results of model validations through permutations. (**C**) Abundance of metabolites in the leverage–SPE scatter plots of the ASCA variables submodels for group (Control vs. Opuntia) and time. T1, T2 and T3 correspond to days 0, 30, and 45, respectively. Metabolomics data analysis was performed using the free web-based metabolomics tool (MetaboAnalyst 4.0; https://www.metaboanalyst.ca/home.xhtml)^54^.

For the time-series metabolomics, an ANOVA-simultaneous component analysis was used to identify the major differences in metabolites between treatments (Control and Opuntia) and major changes in metabolites with time (Table 3 and Fig. 5A). A permutation approach was used to validate the model as verified by significance levels of *P* < 0.01 for both treatment and time (Fig. 5B). Significant variables were identified on the basis of the leverage and squared prediction errors (SPE) associated with each variable, comparing the Control and Opuntia treatments, and comparing times, as shown in Table 3 and Fig. 5C. Treatment effects were identified in five metabolites (2-furanmethanol, diethyl ether, propanal, E-3-octen-2-one, ethanol). As shown in Fig. 5C, several metabolites changed over time in a similar fashion for both the Control and Opuntia treatments, either increasing (Ethanol) or decreasing (2-furanmethanol, 2-octel, methane, bromodichloro-, and perfluronone). Some metabolites remained steady over time: diethyl ether, β-ionone, ethyl butyrate, and heptane in the Opuntia group; propanal and β-pinene in the Control (Fig. 5C). Finally, E-3-octen-2-one concentration decreased over time in the Control group but increased over time in the Opuntia group (Fig. 5C).

**Table 3:** Important features that are well modelled by the ANOVA-simultaneous component analysis (ASCA) variables sub-models for Control and Opuntia sheep, and time.

Group effect (Control vs. Opuntia)	Leverage	Squared prediction errors (SPE)
2-Furanmethanol	0.113	5.78E−33
Diethyl ether	0.081	2.89E−32
Propanal	0.063	1.16E−32
E-3-octen-2-one	0.044	1.16E−32
Ethanol	0.027	7.22E−33
**Time effect**		
Bromodichloro-methane	0.11	0.007
Heptane	0.094	0.024
Ethyl butyrate	0.09	0.053
E- 2-Octenal	0.073	0.033
Beta-ionone	0.067	0.01
2-Furanmethanol	0.056	0.011
Propanal	0.055	0.047
Beta Pinene	0.037	0.001
Ethanol	0.034	0.002
Perfluorononane	0.028	0.001

### Metabolites associated with reproduction

Table 4 presents the correlations among urinary metabolomic compounds and fertility or reproductive rate according to nutritional treatment. Only correlations that were P < 1.0 for either fertility or reproductive rate within a nutritional treatment are presented. From further analysis with Mixed Models of the relationships between each urinary metabolomic compound and fertility or reproductive rate, according to nutritional treatment, it is clear that, in the Control treatment, none of the correlations were significant (P > 0.05). By contrast, in the E-Opuntia treatment, the concentration of 2-methyl-2-propanol, 2-methylbutane and *β*-ionone was positively correlated to fertility rate (P < 0.05). For this treatment, there were no significant correlations between any metabolomic compound and reproductive rate. In the Opuntia treatment, the concentration of demeton-O and *β*-ionone was positively correlated to fertility rate (P < 0.05); whereas the concentration of butane, demeton-O and trimethylamine was positively correlated to reproductive rate (P < 0.05).

**Table 4:** Correlationships among urinary metabolomic compounds and fertility (% pregnant per 100 ewes mated) or reproductive rate (% pregnant per 100 ewes exposed to rams), according nutritional treatment: Control (alfalfa only); Opuntia (Opuntia only) or E-Opuntia (protein-enriched opuntia). Values are *p* value (*r*).

	Fertility	Reproductive rate
**Control**		
Methional	0.06 (0.31)	0.26 (0.18)
**Opuntia treatment**		
Butane	0.13 (− 0.25)	0.06 (− 0.31)
Butanal	0.05 (0.32)	0.18 (0.22)
2,3-Butanediol	0.07 (− 0.30)	0.37 (− 0.16)
Eugenol	0.43 (0.13)	0.09 (0.28)
Glycine, *N*-methyl-*N*-butoxycarbonyl-, propyl ester	0.05 (− 0.33)	0.25 (− 0.20)
Demeton-O	0.001 (− 0.43)	0.05 (− 0.32)
Trimethylamine	0.11 (− 0.26)	0.05 (− 0.31)
Hexane	0.12 (0.26)	0.09 (0.28)
Ethane, 1,1-dichloro-	0.05 (0.32)	0.05 (0.32)
2,4-Octadiene	0.08 (0.28)	0.12 (0.26)
Ethane, 1,1,2,2-tetrachloro-	0.05 (0.32)	0.05 (0.32)
Methional	0.02 (− 0.37)	0.02 (0.38)
3-mercapto-4-methyl-2-pentanone	0.04 (0.32)	0.07 (0.30)
1-Octen-3-ol	0.03 (0.34)	0.11 (0.26)
E-3-octen-2-one	0.03 (0.36)	0.17 (0.24)
epoxy-2-nonenal	0.07 (0.29)	0.13 (0.24)
*β*-ionone	0.01 (0.39)	0.01 (0.38)
**E-Opuntia treatment**		
Ethanol	0.07 (0.30)	0.01 (0.42)
2-Methyl-2-propanol	0.86 (− 0.03)	0.04 (− 0.34)
Ethane, 1,1-dichloro-	0.75 (0.05)	0.06 (0.31)
Methane, bromodichloro-	0.31 (− 0.17)	0.04 (− 0.33)
Ethyl butyrate	0.82 (0.03)	0.05 (0.32)
2-Furanmethanol	0.98 (− 0.004)	0.001 (− 0.52)
Butane, 1,4-dichloro-	0.40 (0.14)	0.04 (0.33)
(2R,4R)-Tetrahydro-4-methyl-2-(2-methylprop-1-enyl)-2H-pyran	0.01 (− 0.40)	0.03 (− 0.35)
Tri-n-butyl phosphate	0.07 (− 0.30)	0.001 (− 0.51)
2-Methylbutane	0.91 (− 0.01)	0.07 (− 0.30)
Heptane	0.26 (− 0.19)	0.04 (− 0.33)
Methane, bromodichloro-	0.37 (− 0.15)	0.09 (− 0.28)
Ethyl butyrate	0.81 (− 0.04)	0.02 (− 0.36)
*β*-Pinene	0.54 (− 0.10)	0.02 (− 0.38)
Epoxy-2-nonenal	0.51 (− 0.11)	0.01 (− 0.41)
*β*-Ionone	0.56 (− 0.09)	0.001 (− 0.55)
Bis(2-methyl-3-furanyl)disulfide	0.12 (− 0.25)	0.03 (− 0.36)

## Discussion

Supplementation with *Opuntia* cladodes, with or without protein enrichment, increased fertility rate and reproductive rate, without any accompanying increases in body weight. The lack of effect on body weight, despite the 48-day duration of treatment, reflects observation in previous studies in two laboratories, with sheep and goats^9,17,19^. The lack of effect on body weight is a critical observation for interpretation of the outcomes for reproduction and fertility because it suggests that the effects of *Opuntia* on the reproductive system do not simply reflect a response to the extra energy provided by supplement and are, in fact, caused by activation of specific metabolomic pathways. Indeed, the metabolomic measurements have revealed pathways that might explain the fertility responses: activation of the metabolism of glucose, methane, tyrosine and glycerolipids.

Positive effects on the growth and developmental competence of preovulatory follicles, with a subsequent improvement in ovulation rate were described in the initial studies showing that cactus cladodes can increase reproductive success in sheep in harsh environments^19,26^. These effects were later confirmed in goats^27,28^. High energy diets increase the concentrations of glucose and metabolic hormones, both of which can act directly on the ovary to enhance the responsiveness of the follicles to follicle-stimulating hormone^29,30^. The responses to *Opuntia* are coherent with this situation because the cladodes are a rich source of soluble sugars^31^ and cladode supplements activate the metabolism of glucose. Specifically, we observed changes in the metabolomic footprints of alcohols (ethanol, 2-methyl-2-propanol; 2-furanmethanol) and gases (propyne, 2R, 4R-tetrahydro-4-methyl-2-2-methylprop-1-enyl-2H-pyran) produced as a result of the fermentation of glucose. We also observed changes in (E,Z)-2,4-heptadienal, a medium-chain aldehyde that is produced during the fermentation of glucose by *E. coli*^32^, perhaps in the rumen^33,34^. Additionally, in grazing lambs, (E,Z)-2,4-heptadienal has been identified in adipose tissue^35^ having apparently been formed from the decomposition of 18:3 n-3 fatty acids^36^. The changes in these processes caused by *Opuntia* supplements suggest that we should look beyond glucose and glucosamine as energy-related factors that affect the ovarian follicles^37,38^.

After correlating each metabolomic compound with either fertility or reproductive rate, we observed that 17 compounds from the E-Opuntia treatment, 17 compounds from the Opuntia treatment and 1 compound from the Control treatment influenced either positive or negative the reproductive variables tested. Yet, a further statistical analysis using mixed models indicated a positive relationship between a few metabolic compounds and fertility rate (2-Methyl-2-propanol, 2-Methylbutane, and β-ionone) in the females from the E-Opuntia treatment. Similarly, within the females from the Opuntia treatment, few metabolic compounds were positively relate to fertility rate (demeton-O, β-ionone) or reproductive rate (demeton-O, butane, trimethylamine). To our knowledge, there are few reports relating these metabolomic compounds with the metabolism of the animal, let alone with reproductive efficiency, so further research is needed to elucidate the underlying mechanism driving these effects.

A positive effect of cactus cladodes on final fertility rates was only found when they were combined with a high-protein supply such as soybean^26^. A reason for this interdependence has not been elucidated but, keeping in mind that the high sugar content of cactus cladodes improves rumen fermentation^39^, a possible hypothesis would be a synergistic interaction between the sugars and a nitrogen source, whether that be urea, soybean or lupin grain. The importance of dietary protein in reproduction in adult female sheep has long been controversial^40,41^ and we suggest that this situation could be resolved by further metabolomic studies.

The sheer diversity of outcomes in metabolomic analysis opens avenues for further research into the relationships between nutrition and reproductive performance in general, not just with *Opuntia*. For example, the metabolomic analysis showed that intake of *Opuntia* cladodes also affected the metabolism of tyrosine and glycerolipids. Tyrosine is essential amino for the production of the thyroid hormones and the catecholamines^42^ that, in turn, influence the activity of GnRH neurons^43^. Tyrosine and lysine are link by hydrogen bonds^44^ and lysine also has a prominent role in cell proliferation^45^ and is therefore important in feto-placental growth during pregnancy^46,47^. Glycerolipids, being mainly triacylglycerides, are used for energy storage and also play a prominent role in pregnancy because they are a major source of energy for the developing fetus following through the provision of fatty acids^48^.

In conclusion, feeding ewes with *Opuntia*, with or without protein-enrichment, can increase the reproductive performance of adult ewes and seems to be a viable option for managing sheep under extensive conditions in arid and semiarid regions. The enhanced reproductive efficiency with *Opuntia* was not accompanied by increases in body weight despite the 34-day duration of nutritional supplementation. This observation, along with the metabolomic analysis of responses to *Opuntia* and protein-enriched *Opuntia*, suggests activation of functional processes that affect the metabolism of glucose, tyrosine, methane, and glycerolipids, all of which could affect the reproductive system through specific processes that do not reflect responses to a simple improvement in feed energy supply. The novelty of these discoveries suggests that we need further research into the mechanisms through which nutrition affects the reproductive system.

## Material and methods

The study was conducted during the breeding period on a commercial farm in northern Mexico (22°15′ N, 100°52 W). All animal procedures were consistent with international guidelines^49^ and with national guidelines^50^ for the care and use of laboratory animals, and met the ARRIVE guidelines for reporting animal research^51^. Our institutional committee approved the experimental study with the reference number 10561934075.

### Experimental design

**Figure 6:**
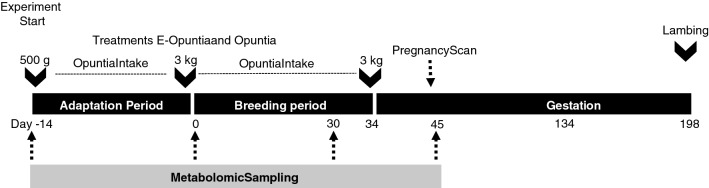
Schema of the experimental protocol. Males were introduced on Day 0 and removed on Day 34. From Day − 14 to Day 0, the female sheep in the E-Opuntia and Opuntia nutritional treatments were allowed to adapt to Opuntia, with the amount offered increasing from 500 g to 3 kg per day. During both the adaptation and breeding periods, sheep in the control treatment received alfalfa at 3% of DM of their body weight.

The experimental protocol is shown in Fig. 6. In August (the breeding season), 60 mature Rambouillet ewes of proven fertility were randomly allocated among three dietary treatments, ensuring similar average body weights for each group: *Opuntia* (Opuntia; n = 20), protein-enriched *Opuntia* (E-Opuntia; n = 20) and a group fed with only alfalfa (Control; n = 20; see details below). The ewes were fed twice daily, at 8–9 a.m. and at 3–4 p.m. *Opuntia* treatments (Opuntia and E-Opuntia) started two weeks before mating (Day − 14) to allow adaptation to the diet and continued until the last day of the mating period (Day 34). Initially, each ewe received 500 g per day and the amount offered was gradually increased during adaptation period to reach 3 kg per animal per day when the experiment started (Day 0). Food refusals were quantified on a pen basis but, after the two first days of the adaptation period, all of the diet offered was consumed. Therefore, on average, the dry matter intake for the Control treatment was 1.3 kg per day for each animal, and for both Opuntia treatments it was 0.78 kg per day for each animal. Before mating; female sheep were dewormed with a commercially available product containing 1 g ivermectin (BAYMEC; BAYER; MEXICO). Additionally, female sheep had received an intramuscular injection of commercially available product containing 0.005 g of B12 (CATOSAL; BAYER; MEXICO) and 500,000 IU of VitA, 75,000 IU of VitD3 and 50 mg of VitE according to manufacturer’s guideline (VIGANTOL; BAYER; MEXICO). Constantly, female sheep had free access to clean water and mineral salts, in blocks containing at least 17% P, 3% Mg, 5% Ca, and 75% NaCl.

On Day 0, the ewes were separated into their treatment groups in three breeding pens, and a single ram with proven libido was introduced into each pen. The rams were rotated through the breeding pens every day until they were removed at the end of Day 34. The ewes were then combined into a single group. Between Days 30 and 45, transabdominal ultrasonography (SAMSUNG-MEDISON SA-600 fitted to a 4 MHz transabdominal convex probe; SAMSUNG CO. Seoul, South-Korea) was used to confirm pregnancy and count the number of fetuses. The ewes were weighed every week throughout the experiment.

### Experimental diets

Control animals were fed alfalfa hay at 3% of their average body mass and treatment groups were fed with *Opuntia* (Opuntia) or protein-enriched opuntia (E-Opuntia) at 3 kg per head per day. *Opuntia* cladodes were harvested daily and cut into small pieces to facilitate consumption. For the E-Opuntia treatment, 600 g urea and 80 g ammonium sulfate were dissolved in 20 L of water, and this solution was sprayed on 100 kg chopped cladodes, after which the material was allowed to stand for 24 h. Diets were provided in a fence-line feeder with sufficient space for all animals to consume all of their respective diet at once. Feed rejection was quantified, but none was rejected after the first few days of the adaptation period. On average, the daily dry matter intake per animal was 1.3 kg for the Control treatment and 0.78 kg for the Opuntia and E-Opuntia treatments. The composition of the diets was analysed by AGROLAB México S.A de C.V (Table 5). All diets provided less than the nutritional requirements for a dry ewe with low physical activity^52^.

**Table 5:** Composition (DM basis) of the diets offered during adaptation, breeding and last trimester of gestation, compared with the requirements for a 50-kg ewe ^52^.

Item	DM (%)	CP (%)	EE (%)	CARB (%)	NDF (%)	ADF (%)	ME (Mcal)
E-Opuntia	26.4	7.3	1.5	41.6	35.8	17.9	2.15
Opuntia	26.7	4.02	2.9	47.1	32	21.5	2.2
Control	93.5	13.9	1.02	19	53	39	1.46
NRC for maintenance	–	9.5	–	–	–	–	2.0

*DM* dry matter, *CP* crude protein, *EE* ether extract, *CARB* carbohydrates, *NDF* neutral detergent fiber, *ADF* acid detergent fiber, *ME* metabolizable energy, *NRC* nutritional requirements of small ruminants^52^.

### Metabolomic assessment

Urine was sampled from a sub-group of ewes from each treatment on four occasions: before the start of treatment (day − 14), during treatment (days 0 and 30) and 10 days after the end of treatment (day 45). For the first sampling, 10 ewes were selected at random from each nutritional treatment and these same 10 ewes were then sampled on every occasion. Urine was collected over ice in sanitized plastic containers with lids and immediately transported to the CIACyT laboratory for storage at − 80 °C until analysis.

Urine samples were analysed by ultrafast gas chromatography with an ‘electronic nose’ (FGC eNose; HERACLES II, ALPHA MOS COMPANY, France) equipped with two columns connected in parallel with different stationary phases, coupled to two ultrasensitive flame ionization detectors (μ-FIDs). Two non-polar MTX 5 columns (10 m × 0.18 mm × 0.4 μm film thickness) and two medium polar MXT-1701 (10 m × 0.18 mm × 0.4 μm film thickness) were used. A subsample (1 mL) of urine was injected in headspace mode under the following conditions: the sample was incubated for 900 s at 40 °C with agitation at 500 rpm; for chromatography, the temperature as initially held at 50 °C for 30 s, after which it was increased isocratically to 280 °C at a rate of 10 °C s^−1^. The temperature of the injector was maintained at 200 °C. The temperature of the detector was maintained at 280 °C. Prior to the chromatographic separation, the 1 mL headspace sample was adsorbed on a Carbowax trap maintained at 60 °C for 50 s while the carrier gas flowed through it in order to concentrate the analytes and to remove excess air and moisture. Subsequently, the sample was desorped by increasing the temperature of the trap to 240 °C in 30 s and the sample was injected. The total separation time was 80 s. Ultrapure hydrogen was used as a drag gas. A standard solution of C6–C16 alkanes was used for the determination of Kovats index. The FGC e‐nose data were processed by Alpha soft V12.44 and AroChembase (Toulouse, France) and VOCs were identified according to the 2011 NIST Retention Index database.

### Statistical analysis

We used the SAS statistical package version 9.3^53^. With linear mixed model procedures (PROC-MIXED) we analyzed the effects of treatment on body weight at the start and end of the breeding period, and on changes in body weight during treatment. Treatment was considered as fixed effect in the model. Body weight at the start and end of the breeding period, and body weight change during the breeding period, were included independently as covariates where appropriate. Body weight values were fitted in a linear regression model (weight on time) for each individual and the regression coefficient was estimated as a measurement of change in weight per unit of time.

Data for fertility rate (percentage of ewes pregnant per 100 ewes mated) was analyzed using the PROC-GLIMMIX with a binomial distribution and logit link function. Treatment was considered as fixed effect. Covariates were body weight at the start and end of the breeding period, and body weight change during the breeding period, and were included independently where appropriate. Data for reproductive rate (number of fetuses in utero per 100 ewes mated) were analyzed using the PROC-GLIMMIX with a multinomial distribution and logit link function. The same fixed effect and covariates were used as for the analysis of fertility. The data for fertility and reproductive rate are presented as logit values and back-transformed percentages.

The correlations among urinary metabolites, body weight change, fertility and reproductive rate during the breeding period were predicted using PROC GLM with the MANOVA option to allow removal of major fixed effects. The fixed effects included in the model were treatment and sampling date.

All two-way interactions among the fixed effect and covariates were included in each model, and non-significant (P > 0.05) interactions were removed from the analysis. Significant differences among means for treatments for variables measured at different time points during the experiment were analyzed using LSD of PROC GLM.

The multivariate statistical analysis of the urinary metabolite data was performed using the web-based metabolomics data processing tool, MetaboAnalyst 4.0^54^. Briefly, for quality Control, variables containing more than 50% missing values (i.e., values lower than limit of detection) were not considered for the statistical analysis. The metabolite data were transformed using the generalized log transformation and then range-scaled to correct for heteroskedasticity, and to reduce mask effects^55^. Partial least squares discriminant analysis (PLS-DA) and variable importance in projection (VIP) were performed using R^56^ to identify the differential metabolites among groups and to rank the metabolites according to their importance in discriminating groups. In addition, PLS-DA was used to differentiate the Control treatment from the Opuntia and E-Opuntia treatments based on the urinary metabolomic data, and to identify the most important metabolites that contributed to the treatment differences. Permutation tests were used to assess the significance of the class discrimination determined by PLS-DA. Classification and cross-validation were performed using the corresponding wrapper function offered by the caret package. In each permutation, a PLS-DA model was built between the data (x) and the permuted class labels (y) using the optimal number of components determined by cross-validation for the model based on the original class assignment. The leverage and the squared prediction error (SPE) were used to evaluate the importance of the metabolite in the model and the fitness of the model for the particular metabolite.

### Ethics approval and consent to participate

All animal procedures were consistent with international guidelines^49^ and with national guidelines^50^ for the care and use of laboratory animals, and met the ARRIVE guidelines for reporting animal research^51^. The experimental study was approved by INIFAP Committee with the reference number 10561934075.

## Supplementary Information

Supplementary Figure 1.

## Data Availability

The data presented in the manuscript are not deposited in an official repository.
