# Supplementary material for: Periconceptional nutrition with spineless cactus (Opuntia ficus-indica) improves metabolomic profiles and pregnancy outcomes in sheep
Source: Sci Rep. 2021 Mar 30;11:7214. doi: 10.1038/s41598-021-86653-w (PMC8010085; doi:10.1038/s41598-021-86653-w)
Supplement: Supplementary file 1 — Supplementary Figure 1. [file 41598_2021_86653_MOESM1_ESM.docx]

**Periconceptional nutrition with spineless cactus (*Opuntia ficus-indica*) improves metabolomic profiles and pregnancy outcomes in sheep**

César A. Rosales-Nieto^1*^, Maribel Rodríguez-Aguilar^2^, Francisco Santiago-Hernandez^3^, Venancio Cuevas-Reyes^4^, Manuel de J. Flores-Najera^5^, Juan M. Vázquez-García^1^, Jorge Urrutia-Morales^3^, Morteza H. Ghaffari^6^,

César A. Meza-Herrera^7^, Antonio González-Bulnes^8^, Graeme B. Martin^9^

*^1^ Facultad de Agronomía y Veterinaria. Universidad Autónoma de San Luis Potosí, San Luis Potosí, 78321, México.*

*^2^ Catedrático CONACYT-Coordinación para la Innovación y Aplicación de la Ciencia y la Tecnología (CIACYT). Universidad Autónoma de San Luis Potosí San Luis Potosí México.*

*^3^ Instituto Nacional de Investigaciones Forestales, Agrícolas y Pecuarias, Campo Experimental San Luis, 78431 San Luis Potosí, México.*

*^4^ Instituto Nacional de Investigaciones Forestales, Agrícolas y Pecuarias, Campo Experimental Valle de México, 56250, Texcoco, Ciudad de México, México.*

*^5^ Instituto Nacional de Investigaciones Forestales, Agrícolas y Pecuarias, Campo Experimental La Laguna, 27440 Matamoros, Coahuila, México.*

*^6^ Institute of Animal Science, Physiology & Hygiene Unit, University of Bonn, 53115 Bonn, Germany.*

*^7^ Unidad Regional Universitaria de Zonas Áridas, Universidad Autónoma Chapingo, 35230, México*

*^8^ Departamento de Producción y Sanidad Animal, Facultad de Veterinaria. Universidad Cardenal Herrera-CEU, CEU Universities. 46115-Alfara del Patriarca, Valencia, Spain.*

*^9^ UWA Institute of Agriculture, University of Western Australia, Crawley, WA 6009, Australia*

*^*^ Corresponding author:* Tel: +524448261314. Email address: [nieto_cesar@hotmail.com](mailto:nieto_cesar@hotmail.com); [cesar.rosales@uaslp.mx](mailto:cesar.rosales@uaslp.mx)

**Supplemental Figure legends**

**Supplemental Figure 1.** Partial least squares-discriminant analysis (PLS-DA) showing 1 cluster for 3 treatments (Control, E-Opuntia, Opuntia) on Day –14. Metabolomics data analysis was performed using the free web-based metabolomics tool (MetaboAnalyst
4.0; <https://www.metaboanalyst.ca/home.xhtml>) [54].

**Supplemental Figure 1.**
